# Supplementary material for: Predicting vaccine effectiveness in livestock populations: A theoretical framework applied to PRRS virus infections in pigs
Source: PLoS One. 2019 Aug 30;14(8):e0220738. doi: 10.1371/journal.pone.0220738 (PMC6716781; doi:10.1371/journal.pone.0220738)
Supplement: S1 Interactive Modelling App — Further details of the model and the interactive app can be found at this link, which is situated at the University of Edinburgh wiki-server, and outlines the components underlying vaccine effectiveness in the field, and their individual and interactive effects. (PDF) [file pone.0220738.s001.pdf]

Supporting information

S1 Interactive modelling app: To determine vaccination strategies to maximise vaccine effectiveness in short and long-term, we developed a user-friendly, interactive modelling app based on our epidemiological model, to predict the epidemiological impact of vaccination with different types of vaccines under different vaccination strategies. The app was developed using R Shiny (R files attached) and the it is accessible via this link: https://vbitsouni.shinyapps.io/Prophylactic_mass_app/

(i) For the effect of different vaccine properties on the dynamis of the model:

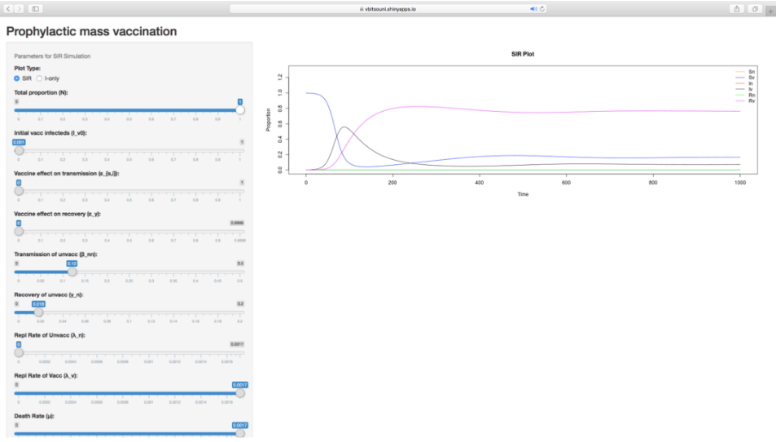

(ii) For the effect of different vaccine properties on the infection dynamics:

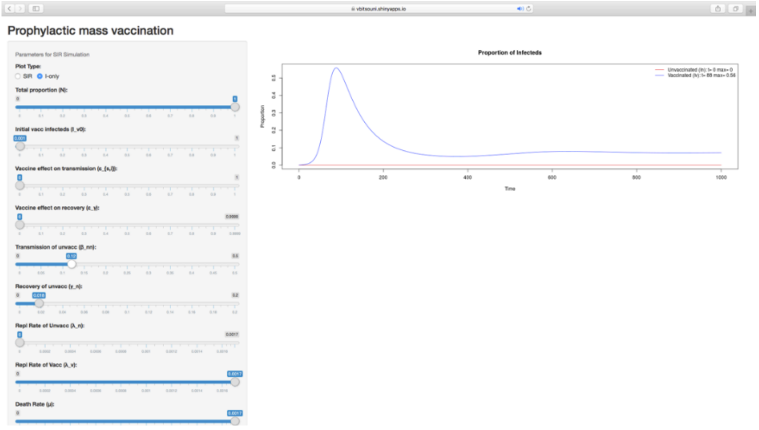

(Non-zero parameters in both screenshots: Initial prop. of vacc. infected animals ($I_V(0)$) = 0.001, Initial prop. of vacc. susceptible animals ($S_V(0)$) = 0.999, Vacc. coverage (p) = 1, Transmission rate between unvacc. animals (β_{NN}) = 0.12, Recovery rate of unvacc. animals (γ_N) = 0.018, Replacment rate of vacc. animals (λ_V) = 0.0017, Death rate (μ) = 0.0017, Death rate due to disease (d) = 0.001.)

Further details of the model and the app can be found at this link: https://www.wiki.ed.ac.uk/display/saphir/, which is situated at the University of Edinburgh wiki-server, and outlines the components underlying vaccine effectiveness in the field, and their individual and interactive effects.
